# Recombinase-based conditional and reversible gene regulation via XTR alleles

**DOI:** 10.1038/ncomms9783

**Published:** 2015-11-05

**Authors:** Camila Robles-Oteiza, Sarah Taylor, Travis Yates, Michelle Cicchini, Brian Lauderback, Christopher R. Cashman, Aurora A. Burds, Monte M. Winslow, Tyler Jacks, David M. Feldser

**Affiliations:** 1 Department of Cancer Biology, Abramson Family Cancer Research Institute and Perelman School of Medicine at the University of Pennsylvania, 421 Curie Boulevard, 751 Biomedical Research Building II/III, Philadelphia, Pennsylvania 19004, USA; 2 Koch Institute for Integrative Cancer Research at the Massachusetts Institute of Technology, Cambridge, Massachusetts 02144, USA; 3 Department of Genetics, Department of Pathology, Stanford University School of Medicine, Stanford, California 94305, USA; 4 The Howard Hughes Medical Institute, Chevy Chase, Maryland 20815, USA

## Abstract

Synthetic biological tools that enable precise regulation of gene function within *in vivo* systems have enormous potential to discern gene function in diverse physiological settings. Here we report the development and characterization of a synthetic gene switch that, when targeted in the mouse germline, enables conditional inactivation, reports gene expression and allows inducible restoration of the targeted gene. Gene inactivation and reporter expression is achieved through Cre-mediated stable inversion of an integrated gene-trap reporter, whereas inducible gene restoration is afforded by Flp-dependent deletion of the inverted gene trap. We validate our approach by targeting the *p53* and *Rb* genes and establishing cell line and *in vivo* cancer model systems, to study the impact of *p53* or *Rb* inactivation and restoration. We term this allele system *XTR*, to denote each of the allelic states and the associated expression patterns of the targeted gene: e*X*pressed (*XTR*), *T*rapped (*TR*) and *R*estored (*R*).

## Abstract (web summary)

Synthetic biology can be used to regulate target genes and uncover gene function in physiologically relevant settings. Here Robles-Oteiza *et al*. describe a new recombinase-based system for conditional inactivation and inducible restoration of gene function and develop new mouse models to study p53 and Rb.

## Graphical abstract

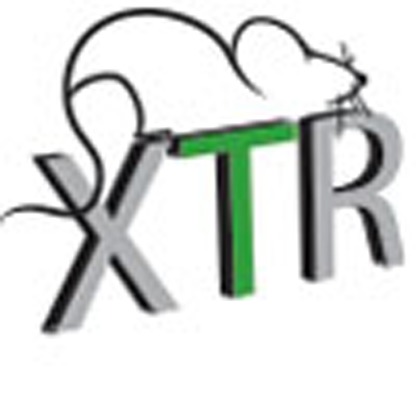

Identifying causal relationships between gene function and the physiological programmes they control is a central goal of biological research and an unmet challenge in cancer. Although loss-of-function mutations in tumour suppressor genes are the most common type of genetic alteration in cancer, identifying how these genes function within physiologically relevant *in vivo* settings remains difficult. Restoration of tumour suppressor gene function has the potential to identify relevant programmes of tumour suppression in physiologically diverse settings. This strategy rests on the premise that latent tumour suppressive programmes are poised to react to the reintroduction of tumour suppressor genes, and that the ensuing changes they orchestrate will expose relevant mechanisms of tumour suppression. In addition, experimental restoration of tumour suppressor genes highlights the potential of future therapies aimed at restoring tumour suppressive pathways to treat cancer. Several different approaches have been used to restore endogenous gene expression *in vivo* and these have identified tumour suppression programmes that are tissue and context specific^1^,^2^,^3^,^4^,^5^,^6^. Despite the power of these approaches, their widespread application to *in vivo* biological systems has been constrained by multiple technological hurdles and limitations inherent to each method (see Discussion).

Conditional approaches to inactivate gene expression in the mouse commonly rely on expression of specialized site-specific recombinases such as Cre and Flp. These recombinases facilitate deletion of DNA sequences that are flanked by similarly oriented DNA elements called loxP or *FRT*, respectively. Cre and Flp can also facilitate the reversible inversion of DNA sequences that intervene inversely oriented loxP or *FRT* sites. However, the inherent reversibility of the inversion reaction is often problematic due to the mosaic pattern of DNA rearrangements that result. To drive reproducible and stable DNA inversions, mutant loxP and *FRT* sites have been used to facilitate the permanent inversion of DNA sequences containing gene traps, to create conditional alleles in mouse embryonic stem (ES) cells and adult mice^7^,^8^,^9^,^10^.

Here we developed a synthetic gene switch, *XTR*, that uses mutant loxP sites to invert a synthetic fluorescent reporter trap element to drive conditional inactivation of endogenous genes. In addition, we engineered the capability to restore accurate endogenous gene expression via Flp-dependent deletion of the synthetic gene switch in a temporally controlled manner. Designed for broad use and compatibility with established recombinase-based tools, XTR alleles provide a powerful method to establish causal relationships between genes and the greater physiological programmes they regulate in specific contexts.

## Results

### XTR allele design

**Figure 1:**
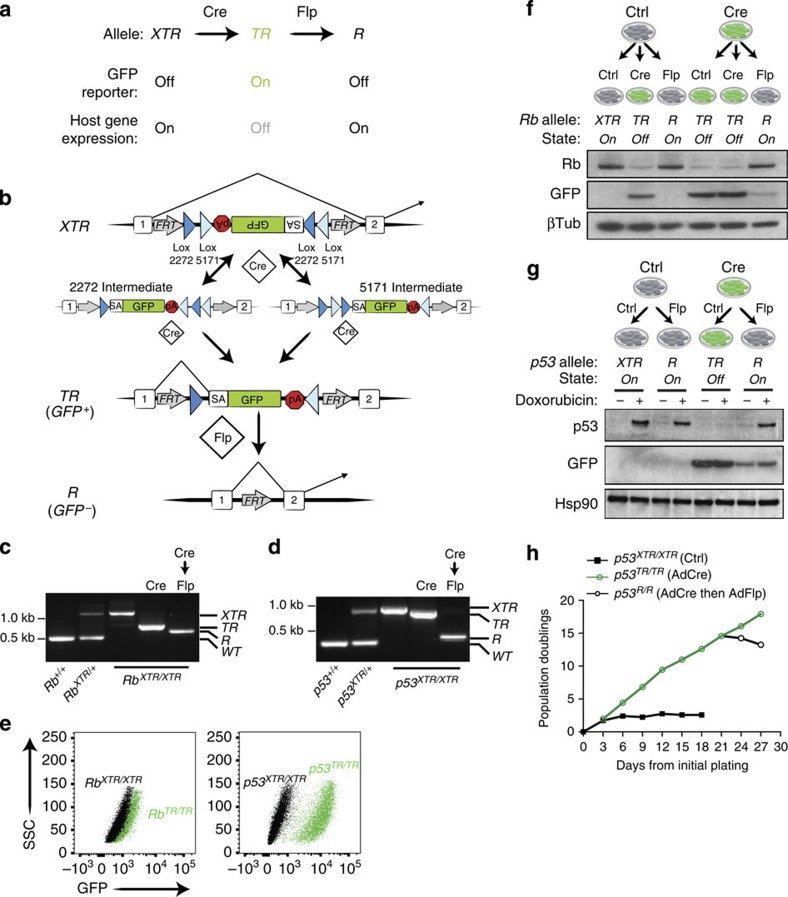
*XTR* alleles facilitate Cre-mediated inactivation and subsequent Flp-dependent restoration of endogenous genes. (**a**) Cre converts *XTR* alleles to the *TR* allele, thereby inactivating gene function. Flp restores gene function by conversion to *R*. (**b**) Schematic of the XTR allele. Cre drives irreversible inversion of a double-floxed gene trap consisting of a splice acceptor (SA) enhanced GFP complementary DNA and the polyadenylation transcriptional terminator sequence (pA). Inversion can proceed either through sequential action of Cre on Lox2272 sites then Lox5171 sites (2272 intermediate) or Lox5171 then Lox2272 sites (5171 intermediate). Stable inversion accepts splicing from upstream exons in the host gene, reads out GFP expression and then terminates transcription, leading to functional inactivation of the host gene’s expression. Flp drives deletion of the gene trap (SA-GFP-pA), thereby restoring normal splicing of the host gene. AdCre and AdCre followed by AdFlpO treatment is indicated (**c**,**d**). PCR-based detection of Rb (**c**) and p53 (**d**) *XTR*, *TR*, *R* and wild-type (*+*) alleles in MEFs of the indicated genotype. (**e**) Detection of GFP reporter expression from *TR* alleles in *Rb*^*TR/TR*^ and *p53*^*TR/TR*^ MEFs by flow cytometry analysis. Representative of ⩾3 cell lines. (**f**) Immunoblot analysis of Rb and GFP expression in *Rb*^*XTR/XTR*^ MEFs treated sequentially with AdCre and/or AdFlpO as indicated. β-Tubulin is a loading control. (**g**) Immunoblot analysis of p53 and GFP expression in *p53*^*XTR/XTR*^ MEFs treated sequentially with AdCre and/or AdFlpO as indicated. Hsp90 is a loading control. (**h**) 3T3 proliferation assay of *p53*^*XTR/XTR*^ MEFs treated sequentially with AdCre (day 3) then AdFlpO (day 21) as indicated. Representative of two *p53*^*XTR/XTR*^ cell lines.

Our goal was to develop a single unifying allelic system that works in concert with existing genetically engineered mouse models of human cancer using the site-specific DNA recombinases Cre and Flp, and would enable temporally controlled gene inactivation followed by accurate and inducible gene restoration (Fig. 1a). To take advantage of the large array of methods to deliver Cre and the diverse model systems that rely on Cre-driven cancer initiation, we developed a ‘double-floxed’ gene trap that can be stably inverted by Cre, to conditionally inactivate virtually any gene of interest. The gene trap consists of a strong adenoviral 40 splice acceptor (SA), followed by the coding sequence for green fluorescent protein (GFP) and the SV40 polyadenylation transcription termination sequence (Fig. 1b). We chose this DNA element because of its small size, ability to report expression from the endogenous host gene once trapped and its proven ability to recapitulate gene knockout phenotypes in cells^11^,^12^,^13^,^14^,^15^,^16^. To enable stable inversion of the gene trap we oriented inverted pairs of Lox5171 and Lox2722 sites in an alternating manner such that each pair flanked the gene trap^9^,^17^. This double-floxed organization results in the permanent inversion of the gene trap after two successive Cre-mediated recombination reactions (Fig. 1b)^7^,^8^. In addition, we flanked the entire gene trap with *FRT* sites to enable deletion of the gene trap by Flp recombinase and restoration of the targeted gene (Fig. 1b). The GFP reporter feature of the XTR system requires in-frame splicing from the upstream exon in instances where XTR is inserted downstream of the translation initiation site of the host gene. To allow general applicability, we developed independent XTR allele targeting vectors with the SA–GFP in each of the three reading frames (Supplementary Fig. 1).

### Targeting and validation of XTR alleles at Rb1 and Trp53

To validate this system and generate alleles that will be of widespread use to the cancer biology community, we targeted the XTR cassette to evolutionarily non-conserved regions of the first intron of the *Trp53* and *Rb1* genes (*p53* and *Rb* hereafter). Correctly, targeted ES cells gave rise to multiple independent mouse lines harbouring *XTR* alleles (Supplementary Figs 2 and 3). To validate each feature of the XTR system, we established murine embryonic fibroblast (MEF) lines from *XTR/XTR*, *XTR/+* and *+/+* littermates for both *Rb* and *p53*. MEF lines were sequentially exposed to Cre and then Flp recombinase via adenoviral infection or through the tamoxifen-inducible Cre-ER fusion protein. In both *p53*^*XTR/XTR*^ and *Rb*^*XTR/XTR*^ MEFs, Cre efficiently converted the *XTR* alleles to the *TR* conformation and Flp subsequently converted the *TR* alleles to the *R* conformation (Fig. 1c,d). A key feature of the XTR system is the ability of *TR* alleles to report host gene expression patterns through GFP expression, while simultaneously eliminating host gene expression. After exposing *p53*^*XTR/XTR*^ or *Rb*^*XTR/XTR*^ MEF lines to Cre, these cells expressed GFP (Fig. 1e). As designed, the pattern of GFP expression reports the activity of the respective promoters for each gene: *p53*^*TR/TR*^ cells expressed robust levels of GFP after Cre-mediated recombination, whereas *Rb*^*TR/TR*^ cells expressed lower yet detectable levels of GFP. Importantly, the induction of GFP in both *p53*^*TR/TR*^ and *Rb*^*TR/TR*^MEFs coincided with a respective loss of p53 and Rb protein expression (Fig. 1f,g). The major goal of the XTR system is to allow a previously inactivated gene to be restored on exposure to Flp recombinase. Infection of *p53*^*TR/TR*^ or *Rb*^*TR/TR*^ MEFs with adenoviral FlpO (mammalian codon-optimized Flp) effectively restored p53 and Rb expression to levels indistinguishable from those in *p53*^*XTR/XTR*^ or *Rb*^*XTR/XTR*^ MEF lines (Fig. 1f,g).

MEFs from wild-type mice lose proliferative potential after multiple serial passages and enter into a senescent state^18^. Consistent with their normal regulation of p53, *p53*^*XTR/XTR*^ MEFs ceased to divide after an initial proliferative phase and entered senescence. To determine whether *TR* alleles phenocopy null alleles, we converted pre-senescent *p53*^*XTR/XTR*^ MEFs to *p53*^*TR/TR*^ MEFs with AdCre. *p53*^*TR/TR*^ MEFs bypassed the proliferative arrest and proliferated indefinitely. Consistent with restoration of *p53* in MEFs using other methods, restoration of p53 expression via AdFlpO treatment in immortalized *p53*^*TR/TR*^ MEF cultures completely arrested cell proliferation, demonstrating that sustained inactivation of p53 is required for cellular immortalization of MEFs (Fig. 1h)^19^. Collectively, these results demonstrate that the *XTR* allele allows normal p53 regulation, the *TR* allele is equivalent to null and the *R* allele restores physiological gene expression.

### *Rb* ^*TR*^ alleles are functionally null

**Figure 2:**
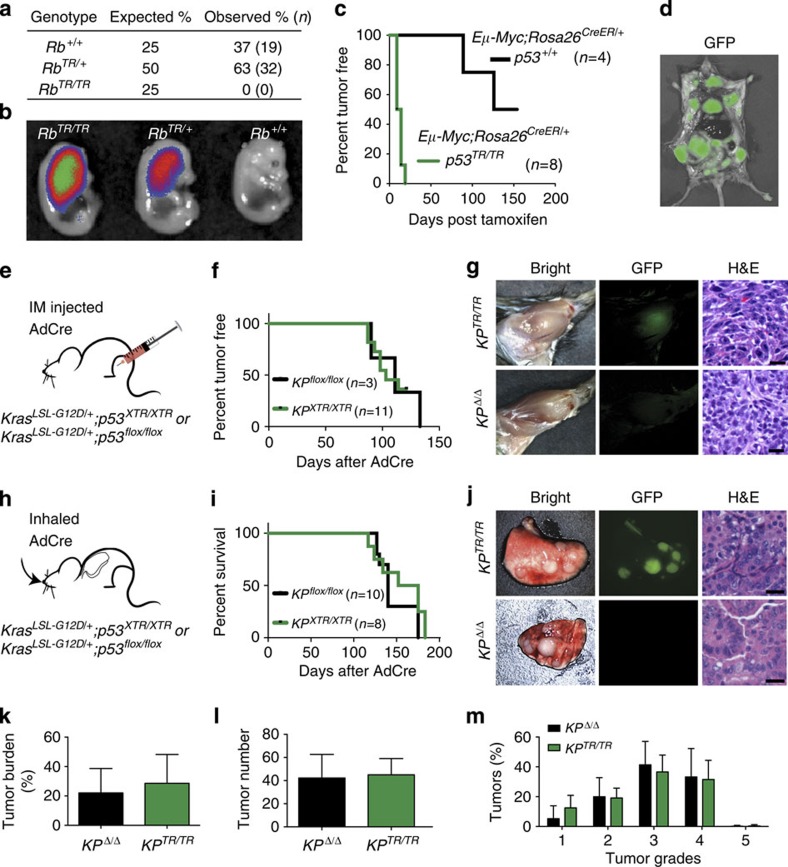
*Rb*^*TR*^ and *p53*^*TR*^ alleles phenocopy conventional floxed and knockout alleles. (**a**) Number of *Rb*^*+/+*^, *Rb*^*TR/+*^ and *Rb*^*TR/TR*^ newborn pubs observed in *Rb*^*TR/+*^X *Rb*^*TR/+*^ crosses. Percentage and number indicated, *χ*^2^=12.4, df=2, *P*=10^−4^. (**b**) GFP detection in *Rb*^*TR/TR*^, *Rb*^*TR/+*^ and *Rb*^*+/+*^ embryonic day 13.5 embryos. (**c**) Kaplan–Meier analysis of lymphoma onset in tamoxifen-treated *Eμ-Myc; p53*^*XTR/XTR*^*; Rosa26*^*CreER/+*^ and *Eμ-Myc; p53*^*+/+*^*; Rosa26*^*CreER/+*^mice; *P*=0.0017, log-rank (Mantel–Cox) test. (**d**) GFP imaging of lymphoma cells in an *Eμ-Myc; p53*^*XTR/XTR*^*; Rosa26*^*CreER/+*^ mouse after lymphoma onset. (**e**) Initiation of sarcomas by intramuscular (IM) injection of AdCre into the hindlimb of *Kras*^*LSL-G12D/+*^*; p53*^*XTR/XTR*^(*KP*^*XTR/XTR*^) and *Kras*^*LSL-G12D/+*^*; p53*^*flox/flox*^(*KP*^*flox/flox*^) mice. (**f**) Kaplan–Meier analysis of sarcoma onset in *KP*^*XTR/XTR*^ and *KP*^*flox/flox*^ mice. (**g**) Representative sarcomas from *KP*^*XTR/XTR*^ (*n*=11) and *KP*^*flox/flox*^ (*n*=3) mice shown by whole-mount bright-field and fluorescent (GFP) microscopy and also (haematoxylin and eosin) staining of histological sections. (**h**) Initiation of lung adenocarcinoma by inhalation of AdCre in *KP*^*XTR/XTR*^ (*n*=8) and *KP*^*flox/flox*^ (*n*=10) mice. (**i**) Kaplan–Meier survival analysis in *KP*^*XTR/XTR*^ and *KP*^*flox/flox*^ mice after inhalation of AdCre. (**j**) Representative lungs from *KP*^*XTR/XTR*^ and *KP*^*flox/flox*^ mice shown by whole-mount bright-field and fluorescent (GFP) microscopy and H&E staining of histological sections. (**k**,**l**,**m**) Comparison of tumour burden (% of lung area), tumour number and tumour grade between *KP*^*XTR/XTR*^ and *KP*^*flox/flox*^mice. Scale bars, 25 μm.

To assess the ability of *Rb*^*TR*^ alleles to functionally inactivate Rb, we crossed *Meox2-Cre* transgenic mice that express Cre in the germline with *Rb*^*XTR*^ animals, to generate *Rb*^*TR/+*^ mice^20^. As *Rb* is an essential gene, we would not expect live-born *Rb*^*TR/TR*^ mice if the *TR* allele phenocopied a knockout allele^21^,^22^. Indeed, out of 51 pups born through these crosses, zero *Rb*^*TR/TR*^ pups were generated, whereas both *Rb*^*+/+*^ and *Rb*^*TR/+*^ pups were observed at the expected frequency (Fig. 2a). Analysis of embryonic day 13.5 mice, a time point before the onset of lethality-causing phenotypes, revealed the presence of grossly normal *Rb*^*TR/TR*^ embryos that expressed GFP. As anticipated, *Rb*^*TR/TR*^ embryos had twofold higher levels of GFP than *Rb*^*TR/+*^ embryos (Fig. 2b). Taken together, these analyses suggest that *Rb* gene function is lost in *Rb*^*TR/TR*^ mice.

### *p53* ^*TR*^ alleles accelerate Myc-driven lymphomagenesis

Loss of p53 expression is causally associated with tumour progression in human cancers and multiple mouse models exist where loss of p53 exacerbates cancer phenotypes. To determine whether XTR alleles could be used to functionally inactivate tumour suppressor genes in diverse tumour models, we crossed the *p53*^*XTR*^ allele to three different well-characterized cancer models. The Eμ-Myc transgenic mouse models Burkitt’s type lymphoma, a B-cell malignancy driven by high levels of Myc expression from the immunoglobulin heavy chain enhancer^23^. Lymphomagenesis in this model is limited by a p53-dependent apoptotic programme and animals deficient in one or both copies of p53 develop aggressive disease within the first month of life compared with 4–6 months in *p53*^*+/+*^ mice^24^,^25^. We exploited the conditional nature of the *XTR* system, to determine whether somatic recombination of *p53*^*XTR/XTR*^ to *p53*^*TR/TR*^ would inactivate p53 expression and lead to rapid onset of lymphoma in Eμ-Myc transgenic mice. *Eμ-Myc; p53*^*XTR/XTR*^*;Rosa26*^*CreER/+*^ and *Eμ-Myc;p53*^*+/+*^*;Rosa26*^*CreER/+*^ mice were aged 10–12 weeks and then treated with a single dose of tamoxifen to activate CreER. Conversion of *p53*^*XTR*^ to *p53*^*TR*^ with tamoxifen treatment led to rapid lymphoma formation with all mice developing aggressive B-cell malignancies with a median onset of 11.5 days. In contrast, *p53*^*+/+*^ mice remained tumour free for prolonged periods after tamoxifen treatment (Fig. 2c). Lymphomas that developed in *Eμ-Myc;p53*^*XTR/XTR*^*;Rosa26*^*CreER/+*^ mice treated with tamoxifen expressed GFP, indicating conversion of *p53*^*XTR*^ to *p53*^*TR*^ and activation of the gene trap. GFP signal was easily detected by direct fluorescence microscopy on necropsy, highlighting the potential utility of the XTR system to track and isolate tumour cells (Fig. 2d).

### *p53* ^*TR*^ alleles phenocopy *p53* ^*flox*^ alleles in cancer models

The XTR system was designed to be compatible with cancer models where additional Cre-dependent alleles are required for tumour formation. To assess this potential, we crossed *p53*^*XTR*^ mice to *Kras*^*LSL-G12D/+*^ mice, which harbour a conditional oncogenic *Kras*^*G12D*^ allele whose expression is dependent on exposure to Cre^26^,^27^. *Kras*^*LSL-G12D/+*^ and traditional *p53*^*flox*^ alleles have been used to model several human tumour types including soft tissue sarcoma and lung adenocarcinoma^28^,^29^. In each of these models, Cre-expressing viruses are delivered directly to the site of tumour formation by direct injection into the muscle to generate sarcomas or inhalation to generate lung adenocarcinoma. To determine whether *p53*^*XTR*^ could work in concert with the *Kras*^*LSL-G12D/+*^ allele and phenocopy *p53*^*flox*^ alleles in these tumour models, we initiated tumours in *Kras*^*LSL-G12D/+*^*p53*^*XTR/XTR*^(*KP*^*XTR/XTR*^) and *Kras*^*LSL-G12D/+*^*;p53*^*flox/flox*^(*KP*^*flox/flox*^) mice and compared the frequency of tumour initiation and degree of histological progression in models of sarcoma and lung adenocarcinoma (Fig. 2e–m). Intramuscular injection of AdCre initiated sarcomas in *KP*^*XTR/XTR*^ and *KP*^*flox/flox*^mice with similar penetrance and median time to sarcoma onset (Fig. 2f). *KP*^*XTR/XTR*^ and *KP*^*flox/flox*^ sarcomas were histologically similar, containing high-grade spindle cell lesions with atypical nuclei and frequent mitotic figures, and consistent with conversion to *p53*^*TR/TR*^ sarcomas arising in *KP*^*XTR/XTR*^ mice were GFP positive (Fig. 2g).

Inhalation of AdCre into *KP*^*XTR/XTR*^ and *KP*^*flox/flox*^ mice resulted in robust lung tumour formation that led to declines in survival with similar rates in each cohort. *KP*^*XTR/XTR*^ mice had tumours with bright GFP signal consistent with conversion to *p53*^*TR/TR*^ (Fig. 2j). Tumour number, grade and the extent of tumour burden in *KP*^*flox/flox*^ and *KP*^*XTR/XTR*^ mice were also indistinguishable (Fig. 2k–m). Taken together, these data demonstrate that XTR is a robust method to inactivate tumour suppressor gene function in diverse tumour types with similar effectiveness to conventional floxed alleles. In addition, the bright GFP signal from the *p53*^*TR/TR*^ allele affords a convenient method to identify, isolate and track tumour cells.

### FlpO-ER facilities efficient restoration of *p53* ^*TR*^ alleles

**Figure 3:**
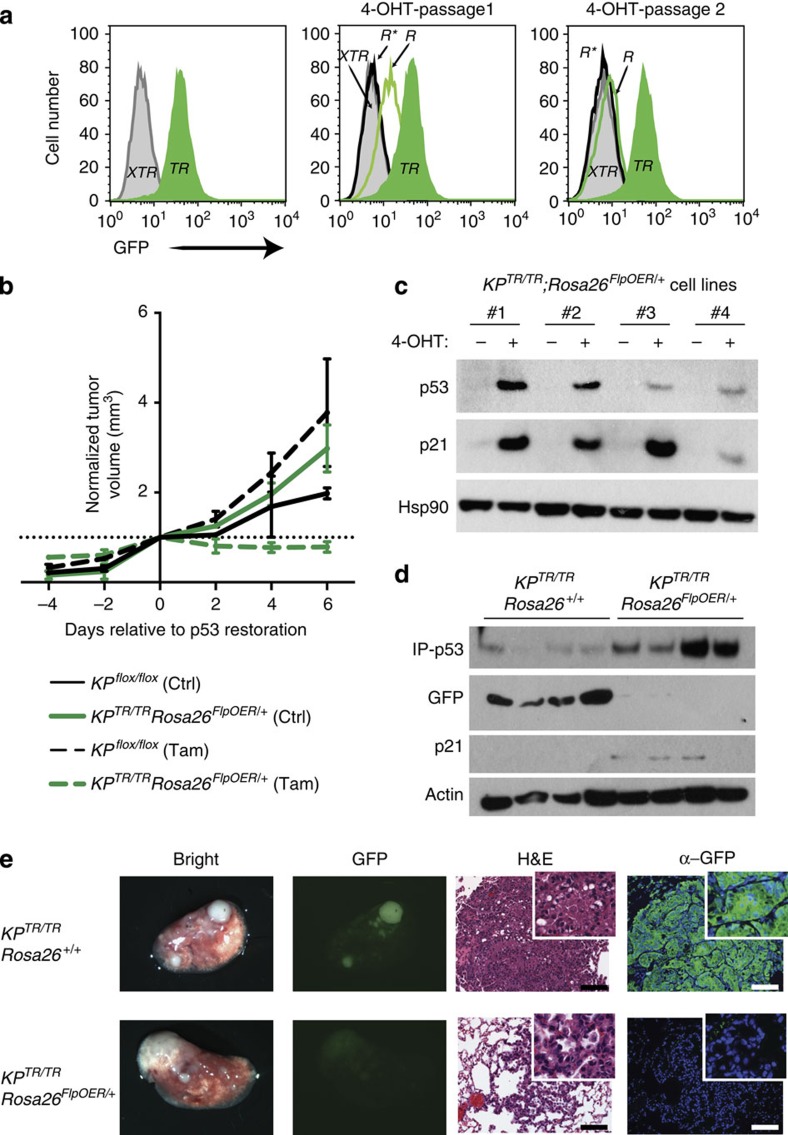
Tamoxifen-regulated FlpO-ER efficiently restores *p53*^*TR*^ alleles to *p53*^*R*^*in vitro* and *in vivo*. (**a**) GFP detection by flow cytometry analysis of *KP*^*XTR/XTR*^*; Rosa26*^*FlpO-ER/+*^ MEFs. Untreated (*p53*^*XTR/XTR*^: solid grey), AdCre treated (*p53*^*TR/TR*^: solid green), tamoxifen-treated cells previously untreated (*p53*^R*/R*^: open black trace) or previously AdCre-treated (*p53*^*R/R*^: open green trace). R*, direct conversion of *p53*^*XTR*^ to *p53*^*R*^. (**b**) Analysis of transformation potential of *KP*^*XTR/XTR*^*; Rosa26*^*FlpO-ER/+*^ MEFs after *in vitro* AdCre treatment followed by subcutaneous engraftment into nude mice. Tumour growth monitored by caliper measurements at the indicated times. Tamoxifen was administered on day 0, 1 and 2 to all mice. Representative of two cell lines (*n*=4). (**c**) Immunoblot analysis of lysates from *KP*^*TR/TR*^*; Rosa26*^*FlpO-ER/+*^ lung tumour-derived cell lines 24 h after addition of 4-hydroxytamoxifen (4-OHT) or vehicle control. (**d**) Immunoblot analysis of lysates from micro-dissected lung tumours 7 days after tamoxifen delivery. Immune precipitation of p53 was required before western blotting for detection. (**e**) Microscopic analysis of lung tumours derived from *KP*^*XTR/XTR*^*; Rosa26*^*FlpO-ER/+*^ and *KP*^*XTR/XTR*^*; Rosa26*^*+/+*^ mice 7 days post tamoxifen treatment. From left to right, bright-field and fluorescence stereo microscopy, haematoxylin and eosin staining and immunohistological staining for GFP in representative tumours. Scale bars, 100 μm.

Robust methods to temporally regulate Flp activity *in vivo* are critical to realize the full potential of the XTR system. Based on our success using oestrogen receptor (ER) fusions to Cre recombinase in regulating loxP-directed recombination events *in vivo*^2^, we generated *KP*^*XTR/XTR*^*;Rosa26*^*FlpO-ER/+*^ mice that ubiquitously express a tamoxifen-inducible FlpO-ER fusion^30^. *KP*^*XTR/XTR*^*;Rosa26*^*FlpO-ER/+*^ MEFs were treated with AdCre to recombine *p53*^*XTR/XTR*^ to *p53*^*TR/TR*^ and monitored by flow cytometry for GFP expression. After 3 days, nearly all of the AdCre-treated cells were GFP positive as expected (Fig. 3a). On replating, we treated cells with 4-hydroxytamoxifen to activate FlpO-ER activity and monitored GFP expression by flow cytometry. Consistent with the long half-life of GFP, at the first passage GFP expression was reduced fivefold in all cells and by the second passage GFP levels had diminished to background levels (Fig. 3a). Consistent with cooperative effects of Kras and p53 mutations to drive transformation, *KP*^*TR/TR*^*;Rosa26*^*FlpO-ER/+*^ MEFs rapidly formed tumours after subcutaneous transplantation in nude mice. Importantly, tamoxifen treatment of mice bearing *KP*^*TR/TR*^*;Rosa26*^*FlpO-ER/+*^ tumours resulted in rapid loss in growth potential but had no effect on *KP*^*flox/flox*^ tumours (Fig. 3b).

To assess the capabilities of FlpO-ER to regulate tumour suppressor gene restoration in the context of an autochthonous cancer model, we initiated tumours in the lungs of *KP*^*XTR/XTR*^*;Rosa26*^*FlpO-ER/+*^ mice by inhalation of AdCre. Initially, we derived cell lines from individual *KP*^*TR/TR*^*;Rosa26*^*FlpO-ER/+*^ tumours. *In vitro* administration of 4-hydroxytamoxifen led to robust induction of p53 and the p53 target gene p21, consistent with FlpO-ER-mediated conversion of *p53*^*TR/TR*^ to *p53*^*R/R*^(Fig. 3c). Importantly, tamoxifen treatment of *KP*^*XTR/XTR*^*;Rosa26*^*FlpO-ER/+*^ mice 13 weeks post tumour initiation also lead to efficient conversion of *p53*^*TR/TR*^ to *p53*^*R/R*^. Tamoxifen treatment lead to upregulation of p53 and p21, and a concomitant loss of GFP specifically in *KP*^*TR/TR*^*;Rosa26*^*FlpO-ER/+*^ but not *KP*^*TR/TR*^*;Rosa26*^*+/+*^ tumours (Fig. 3d). In addition, direct fluorescence microscopy of harvested lungs or immunohistological analyses of lung tumour sections revealed that *KP*^*TR/TR*^*;Rosa26*^*+/+*^ control tumours displayed multiple GFP-positive tumours in the lung, suggesting that they maintained *p53*^*TR/TR*^ alleles. In contrast, tamoxifen treatment of *KP*^*TR/TR*^*;Rosa26*^*FlpO-ER/+*^ tumour-bearing mice resulted in tumours that had dim or undetectable GFP levels, indicating efficient conversion to *p53*^*R/R*^ alleles (Fig. 3e).

## Discussion

Restoration of tumour suppressor gene function in cancer cells *in vivo* has proven to be a powerful means to identify context-specific programmes of tumour suppression. However, the widespread practice of restoring gene function in established tumours within their natural setting has been greatly limited by previous approaches that are incompatible with specific genes of interest or by strategies that require multiple technically challenging steps to implementation^1^,^2^,^3^,^4^,^5^,^31^.

We and others have used genetically engineered alleles in which a loxP-flanked transcription/translation stop cassette (loxP-STOP-loxP; LSL) is inserted into the first intron of a gene of interest^1^,^2^. An LSL allele is a null allele until Cre-mediated recombination deletes the STOP cassette, thus allowing normal expression of the targeted genes. For example, the *p53*^*LSL*^ allele is a functionally null allele of p53 and *p53*^*LSL/LSL*^ mice develop spontaneous lymphomas and sarcomas at the same frequency and rate as p53 KO mice^1^. Initially, *p53*^*LSL/LSL*^ mice were used to study the consequences of p53 restoration in T-cell lymphomas and soft tissue sarcomas that naturally arise in p53-deficient mice^1^. Most genetically engineered mouse cancer models rely on Cre to activate or inactivate genes of interest, but because LSL approaches require Cre for gene restoration they are not compatible with these existing models. Because of this major limitation, we made use of a lung cancer model (*Kras*^*LA2/+*^) where *Kras*^*G12D*^ is activated spontaneously due to a stochastic recombination event^32^. These mice develop lung adenocarcinomas with 100% penetrance at an early age. This afforded us the opportunity to generate *Kras*^*LA2/+*^*;p53*^*LSL/LSL*^ mice and to restore p53 in these early-to-moderate stage lung cancers^2^. Performing this restoration in large cohorts of mice was frustrating, owing to the mortality associated with the frequent and rapid development of sarcomas and lymphomas in p53-deficient mice. Extensive ageing of the mice was not possible and we were unable to assess the effects of p53 restoration on the most clinically relevant advanced stages of primary lung tumours and metastases.

The most critical limitation of the LSL system is that LSL alleles cause germline deficiency. Thus, LSL alleles cannot be used to study genes that are required for embryonic development. Unfortunately, the vast majority of tumour suppressor genes are embryonic lethal (for example, *Rb1*, *Pten*, *Apc*, *Nf1*, *Nf2*, *Ptc*, *Vhl*, *Smad4*, *Atr*, *Smarca4*, *Arid1a*, *Snf5*, *Nkx2-1*, *Nkx3-1*, *Tsc1*, *Tsc2* and so on), thus leaving very few tumour suppressor genes with which to use this system (for example, *p53*, *Cdkn2a* and *Atm*). This fact, together with its incompatibility with other Cre-based systems, has severely limited the utility of this approach.

Fusion of an ER fragment to proteins may, in some instances, allow for tamoxifen-dependent activity of the fused protein. In the limited cases where this has proven effective, it has been a robust method. A *p53*^*ERTam*^ knock-in allele has been used to model p53 restoration in Myc-induced lymphomas and in *Kras*^*G12D*^-induced lung adenocarcinomas^3^,^4^. Although, unlike the LSL approach, the ER-fusion alleles are compatible with Cre-based cancer models, ER fusions are still limited to non-essential genes, as mice with homozygous knock-in would still be expected to recapitulate the embryonic lethality of null mice. In addition, not all proteins will tolerate carboxy and/or amino-terminal fusions with the ER and there can be concern of unknown alterations in function of the ER fusion protein. Finally, this approach is limited to proteins that carry out their functions in the nucleus, as the mechanism of induction is based on tamoxifen-induced nuclear translocation of the fusion protein.

Regulatable small hairpin RNA (shRNA) is a very different method that overcomes several of the limitations associated with LSL and ER fusion-based approaches. However, the techniques involved are challenging and, to date, the generation of regulatable RNA interference transgenic mice has been employed by few laboratories. This approach has been used to regulate the expression of three tumour suppressor genes (*Apc*, *Pten* and *p53*) in relevant cancer models^5^,^6^,^31^,^33^,^34^. To effectuate potent knockdown of the target gene and recreate phenotypes equivalent to null alleles, multiple specialized techniques, mouse strains and ES cell lines are required^35^. The success of the regulatable shRNA to recapitulate phenotypes associated with gene loss requires the ability to generate a sufficiently potent shRNA. Screening of dozens of shRNAs is therefore required and specialized protocols to ascertain whether an shRNA is likely to be effective as a single-copy integrated transgene is necessary^35^,^36^. Despite this, potent shRNAs have been identified that approximate null alleles in certain experimental systems^5^,^31^,^37^,^38^.

Off-target effects associated with shRNA expression are also potentially problematic^39^. High expression of a heterologous shRNA could obscure biological readouts by knocking down the expression of unintended messenger RNA targets or by overwhelming the RNA interference processing machinery to such an extent that naturally expressed micro RNAs are not normally produced^40^,^41^,^42^. Each of these technical issues may have profound consequences on the biology of cells in question that will have an impact on data interpretation.

Currently, regulatable shRNA strategies rely on tet-based systems which limits their functionality within other systems that also utilize tet-regulated transgenes. For example, multiple cancer models rely on tet-inducible oncogenes to drive tumour formation and these models rely on continual expression of the oncogenic driver to maintain the cancer^43^,^44^,^45^,^46^. Although the regulated shRNA can be easily added into this approach to ascertain the added effect of target gene knockdown, the ability to cleanly determine the effect of target gene restoration is not possible due to the simultaneous loss of oncogene expression on doxycyclin removal.

The simplicity and functionality of the XTR allele system offers several significant advantages over existing strategies, to interrogate gene function in the mouse. Similar to conventional approaches to create conditional alleles in the mouse, XTR integration relies on gene targeting in ES cells. Creation of XTR alleles uses the same methods that are standard protocols in academic and commercial ES cell/transgenic mouse facilities and thus requires no specialized technical hurdles. As outlined in Supplementary Fig. 3, generation of XTR alleles requires a *neoXTR* allele intermediate that necessitates secondary selection of either ES cell clones or pups that lost the FRT-Neo-FRT. For p53 and Rb, this was achieved by either electroporation with Flp-expressing plasmids or by crossing with germline Flp-expressing mice^47^. Further use of the XTR system at other loci will be necessary to determine whether locus-dependent effects exist that could limit this strategy. CRISPR-based approaches to generate conditional alleles by directly injecting zygotes with modified XTR vectors lacking the FRT-Neo-FRT cassette could obviate the need for this step^48^,^49^.

XTR combines three separate tools into one discrete genetic element to conditionally inactivate a gene of interest, accurately report host gene expression once inactivated and facilitate precise gene restoration in an inducible manner. Bringing these tools together into one strategy offers unparalleled functionality to a single genetically engineered allele. With the increased functionality to mark gene inactivation and report accurate gene expression levels of the targeted gene, as well as the ability to rescue gene function, XTR is positioned to greatly expand current capabilities to interrogate gene function within *in vivo* systems. Thus far, our experience targeting XTR to *p53* and *Rb* loci suggest a ‘plug and play’ simplicity that abrogates the need for development, testing and optimization that is associated with ER fusion and regulatable shRNA strategies. However, targeting of additional loci will be required to affirm the generalizable nature of the XTR approach with respect to preserving proper host gene regulation and robustness of gene inhibition. Although the XTR system would not be compatible with the few examples of Flp-dependent alleles that exist^50^,^51^, its seamless integration into the numerous Cre-based model systems available should facilitate its widespread utility. Similar to most conditional approaches to inactivate gene function in the mouse, XTR alleles require specialized methods or mouse strains to deliver Cre recombinase to specific cell types of interest. Our data suggest that either promoter-specific transgenes or viral based approaches to deliver Cre to tissues of interest is a robust strategy to inactivate genes of interest in the mouse with XTR. Finally, restoration of gene function using XTR alleles requires strategies to regulate Flp activity. As demonstrated, we used a tamoxifen-inducible *Rosa26*^*FlpO-ER*^ allele that is widely expressed and therefore suitable for a broad range of applications using the XTR system^30^. However, additional strategies to regulate FlpO may augment the utility of the XTR system in specialized scenarios.

Here we have targeted the XTR cassette to two important tumour suppressor genes to address cancer-relevant questions. However, we envision XTR as a powerful approach to investigate gene function in diverse biological settings to gain important insight into mechanisms at the tissue, cellular or molecular level. In addition, XTR alleles have the potential to model therapeutic interventions in disease settings, where temporarily inactivating a putative drug target through the genetic means intrinsic to XTR could predict efficacy or identify unforeseen complications of future therapies. More broadly, the ability to restore gene function using the XTR system offers a major opportunity in conditional genetic methods to facilitate the widespread application of *in vivo* gene restoration approaches.

## Methods

### Creation of base targeting vectors

The XTR allele requires targeting to the desired gene loci, to carry out inducible gene trapping via splice acceptance from upstream exons; therefore, *pNeoXTR* plasmids were created to accept splicing from each of the three reading frames (Supplementary Fig. 1; pNeoXTR f0 (Addgene #69157), pNeoXTR f1 (Addgene #69158) and pNeoXTR f2 (Addgene #69159)). Components were assembled from pL451 (Addgene #22687), pFLIP-FF^9^ (a gift from Patrick Stern) and pSA-GFP-pA^11^ (gift from Jan Carette). Alternative reading frames were established by Quickchange method (Agilent) following the manufacturer’s instructions. Full sequence of each targeting plasmid is available at Addgene. Targeting arms were generated by PCR and cloned into base targeting vectors. Amplification of the p53 left arm was accomplished with forward primer 5′- tggcgcgccggatcccagcactactgtggttaag -3′ and reverse primer 5′- ttgcggccgcagaggtttgagtacaaccagggctg -3. Amplification of the p53 right targeting arm was accomplished with forward primer 5′- gtgttaattaacgagtctattgcctttcccagccaac -3′ and reverse primer 5′- ctgcagccaaaggtccagttacagg -3′. Amplification of the Rb left arm was accomplished with forward primer 5′- cacggcgcgccgtcaaacagctatgaccatg -3′ and reverse primer 5′- ccagcgatcgcctcgagaacttaatgatggg -3′. Amplification of the Rb right arm was accomplished with forward primer 5′- gtggttaattaaggctcgag -3′ and reverse primer 5′- gtgttaattaaaaagggatgcaaatagaagg -3′.

### ES cell culturing and electroporation

Targeting constructs were linearized and electroporated independently into F1 C57BL/6 × 129S4 hybrid v6.5 ES cells (gift from Rudolph Jaenisch) using standard conditions. Neomycin (300 μg ml^−1^ G418)-resistant colonies were isolated, expanded and screened by PCR and Southern blotting. Correctly targeted clones were either directly injected into C57BL/6J blastocysts or first electroporated with supercoiled pCAGGS-FLPe (gift from R. Jaenisch) and plated, to generate subclones. ES cell subclones lacking the NeoR cassette after FLP recombination were then injected into C57BL/6J blastocysts. Both strategies yielded several high-percentage chimeras and the establishment of the mouse lines.

### Identification of targeted ES clones

PCR was used to screen for probable targeted clones for both p53 and Rb NeoXTR alleles. Reactions spanning the left targeting arm for p53NeoXTR-targeted clones identified positive clones with primers 5′- ttcaagagacggagaaagggcg -3′ and 5′- tggatgtggaatgtgtgcgag -3′. These were subsequently screened via Southern blotting. PCR screening strategies for Rb were not successful and failed to detect even those subsequently identified by Southern blotting. For Southern blotting, genomic DNA was digested overnight at 37 °C with 20 Units of each restriction enzyme. The next day, another 10 Units were added and incubated at 37 °C for an additional 4–6 h. DNA fragments were resolved on 0.7% tris-acetate-EDTA gels at low voltage overnight, stained with ethidium bromide, depurinated, denatured and neutralized before transfer to Hybond XL membranes. Ultraviolet cross-linked DNA was hybridized with radioactive probes suspended in Express Hyb solution (Clontech) as per the manufacturer’s instructions. Radiolabelled probes were generated by random primer methods using PrimeIT II Kits (Agilent) and purified with Quick Spin Columns (Roche). DNA probes were generated by PCR of mouse genomic DNA. Rb 5′-probe was amplified with 5′- attaagttctcgattcctcag -3′ and 5′- tgccaggcggacccgactttg -3′ primers. The p53 3′-probe was amplified with 5′- atagtgggaaccttctgggacg -3′ and 5′- cagtggaggagcacctgtcttatg -3′ primers. The GFP probe was generated using pSA-GFP-pA as a template with 5′- atggtgagcaagggcgagga -3′ and 5′- ttacttgtacagctcgtcca -3′ primers. Genomic DNA from ES cell clones following Flpe electroporation was screened by PCR with the following primers to identify a 528-bp product (*Rb*^*XTR*^) or a 587-bp product (*p53*^*XTR*^): p53 5′- caactgttctacctcaagagcc -3′ × 5′- taaaaaacctcccacacctcccc -3′ and Rb 5′- tgttgttattgtcagcactag -3′ × 5′- TAAAAAACCTCCCACACCTCCCC -3′.

### Germline deletion of *Neo* ^*R*^

*p53*^*neoXTR/+*^ and *Rb*^*neoXTR/+*^ mice were crossed to *Rosa26*^*FlpeR/+*^ mice. *p53*^*neoXTR/+*^*; Rosa26*^*FlpeR/+*^ and *Rb*^*neoXTR/+*^*; Rosa26*^*FlpeR/+*^ pups were found to contain all possible alleles after Flp reactions (*neoXTR*, *XTR* and *R*), and were therefore always considered to be mosaic. *p53*^*neoXTR/+*^*; Rosa26*^*FlpeR/+*^ and *Rb*^*neoXTR/+*^*; Rosa26*^*FlpeR/+*^ mice were subsequently crossed to wild-type mice and pups that lost the *Rosa26*^*FlpeR*^ allele were screened for retention of XTR and loss of *Neo*^*R*^. See also Supplementary Fig. 3.

### Generation and analysis of MEFs

MEFs were generated by timed matings from E13.5 to E15.5 embryos. Adenoviral infections to express Cre or FlpO recombinases (purchased from Gene Transfer Vector Core at the University of Iowa) were carried out on sub-confluent cultures using 10^7^–10^8^ viral particles in 6- or 10-cm dishes. Quantification of GFP in MEFs was performed on an LSR II cytometer (BD Biosciences) at the PennFlow Core. Proliferation assays were carried out in 3T3 format counting and replating 3 × 10^5^ cells every 3 days in 6-cm dishes. Genotypes of MEFs were analysed via three-primer PCR reactions using purified genomic DNA as a template to detect *XTR*, *TR*, *R* and *WT* alleles. Primers used for *Trp53* were as follows: (1) 5′- cttggagacatagccacactg -3′, (2) 5′- caactgttctacctcaagagcc -3′ and (3) 5′- cttgaagaagatggtgcg -3′. Primers used for *Rb1* were as follows: (1) 5′- tgttgttattgtcagcactag -3′, (2) 5′- ggcagaggcagtaaaacagagagc -3′ and (3) 5′- cttgaagaagatggtgcg -3′.

### Immunodetection in cultured cells

Cells were lysed in RIPA buffer and resolved on NuPage BT gels and then transferred to polyvinylidene difluoride (Millipore). Blottings were probed with the following antibodies: p53 (Novocastra IMX25, western, 1:500) or Santa Cruz (FL-393, IP 1 μg), Rb (Santa Cruz, C-15, 1:500), p21 (Santa Cruz, F-5, 1:500), GFP (Cell Signaling Technologies, #2956, 1:1,000), Hsp90 (BD Transduction Labs, Clone 68, 1:1,000), β-Tubulin (Cell Signaling Technologies, #2146 1:5,000), Actin (Sigma, A2066, 1:1,000). Immunoprecipitation was carried out in RIPA buffer with standard techniques using Protein G beads. 4-Hydroxytamoxifen dissolved in ethanol was administered once at the time of cell plating at a final concentration of 500 nM. AdCre or AdFlpO (5 × 10^7^ pfu) was purchased from the University of Iowa Gene Vector Core and administered to adherent cells at the indicated times. Full versions of blots are shown in Supplementary Fig. 4.

### Tumour-derived cell lines

Tumours were excised from the lungs of mice using dissecting stereo microscopy, dissociated with Collagenase IV and trypsin for 30–60 min, then quenched with fetal bovine serum, washed in Hank’s balanced salt solution, then cultured in high glucose-containing DMEM supplemented with 10% fetal bovine serum and antibiotics at 37 °C and 5% CO_2_ until cell line establishment (∼2–4 weeks).

### Animal studies and treatments

All animal experiments were carried out under strict compliance with Institutional Animal Care and Use Committee at either MIT or Penn. for *Kras*^*LSL-G12D*^ (ref. 26), *p53*^*flox/flox*^ (ref. 52), *Rosa26*^*FlpeR*^ (ref. 47), *Rosa26*^*FlpO-ER*^ (ref. 30) and *Meox2-Cre*^20^ mice have been described. Sarcoma and lung adenocarcinoma inductions were carried out as described^29^,^53^. Tumour grading was performed at the PennVet Comparative Pathology Core using established tumour-grading schemes^28^,^29^. Tamoxifen treatment was performed on 3 consecutive days via oral gavage of 200 μl of a 20 mg ml^−1^ solution dissolved in 90% sterile corn oil and 10% ethanol.

### Histology and microscopy

Fluorescent signals from tumours were imaged with either the IVIS Spectrum (Caliper Life Sciences) or a fluorescence stereo dissection microscope (Leica). Tissues were dissected into 10% Neutral buffered formalin and fixed 16–20 h at 4 °C before being dehydrated in a graded alcohol series. Paraffin-embedded histological sections were produced at the Abramson Family Cancer Research Institute Histopathology Core as 4 μm sections before staining. Immunostaining was carried out after citrate-based antigen retrieval with α-GFP antibody (Abcam ab13970, 1:1,000) and fluorescent secondary detection (Thermo Fisher, α-Chicken Alexa Fluor 594, 1:200). Microscopy was performed on a Leica DMI6000B inverted light and fluorescent microscope.

## Additional information

**How to cite this article:** Robles-Oteiza, C. *et al*. Recombinase-based conditional and reversible gene regulation via XTR alleles. *Nat. Commun.* 6:8783 doi: 10.1038/ncomms9783 (2015).

## Supplementary Material

Supplementary Information Supplementary Figures 1-4
