# Supplementary material for: Recombinase-based conditional and reversible gene regulation via XTR alleles
Source: Nat Commun. 2015 Nov 5;6:8783. doi: 10.1038/ncomms9783 (PMC4635517; doi:10.1038/ncomms9783)
Supplement: Supplementary Information — Supplementary Figures 1-4 [file ncomms9783-s1.pdf]

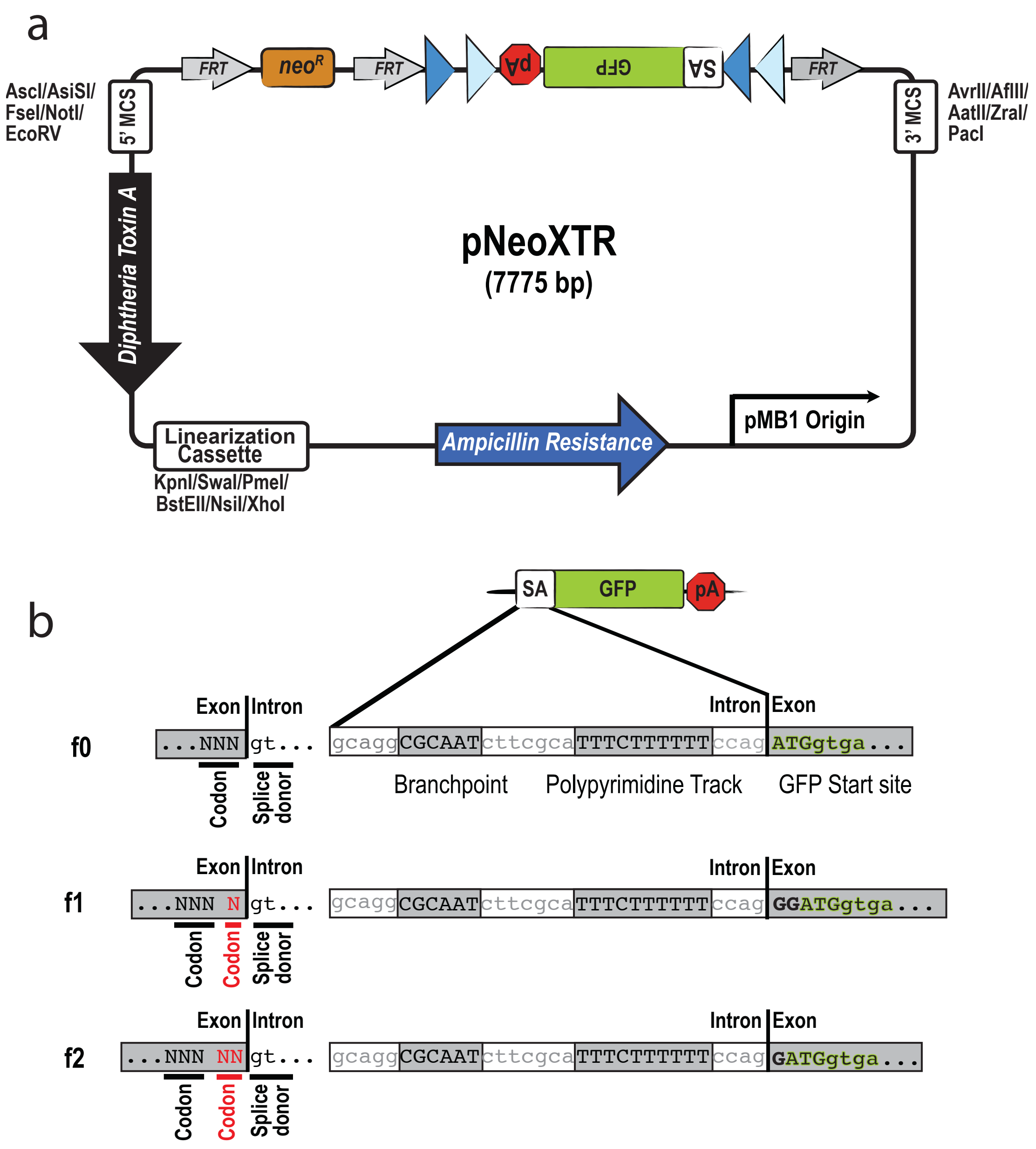

Supplementary Fig. 1 pNeoXTR Targeting vector design. (**a**) pNeoXTR Plasmid map. Multiple cloning sites (MCS) and unique restriction enzyme sites are indicated for cloning of 5' and 3' targeting arms. Unique restriction sites indicated for the linearization cassette. (**b**) Three separate pNeoXTR vectors (f0, f1, and f2) were designed to accept splicing in each of the three reading frames. The f0, f1, and f2 vectors are designed to accept splicing from an upstream intron that would donate 0, 1, or 2 nucleotides (red) to the next exon respectively. Any pXTR vector could be used to target a non-coding upstream exon.

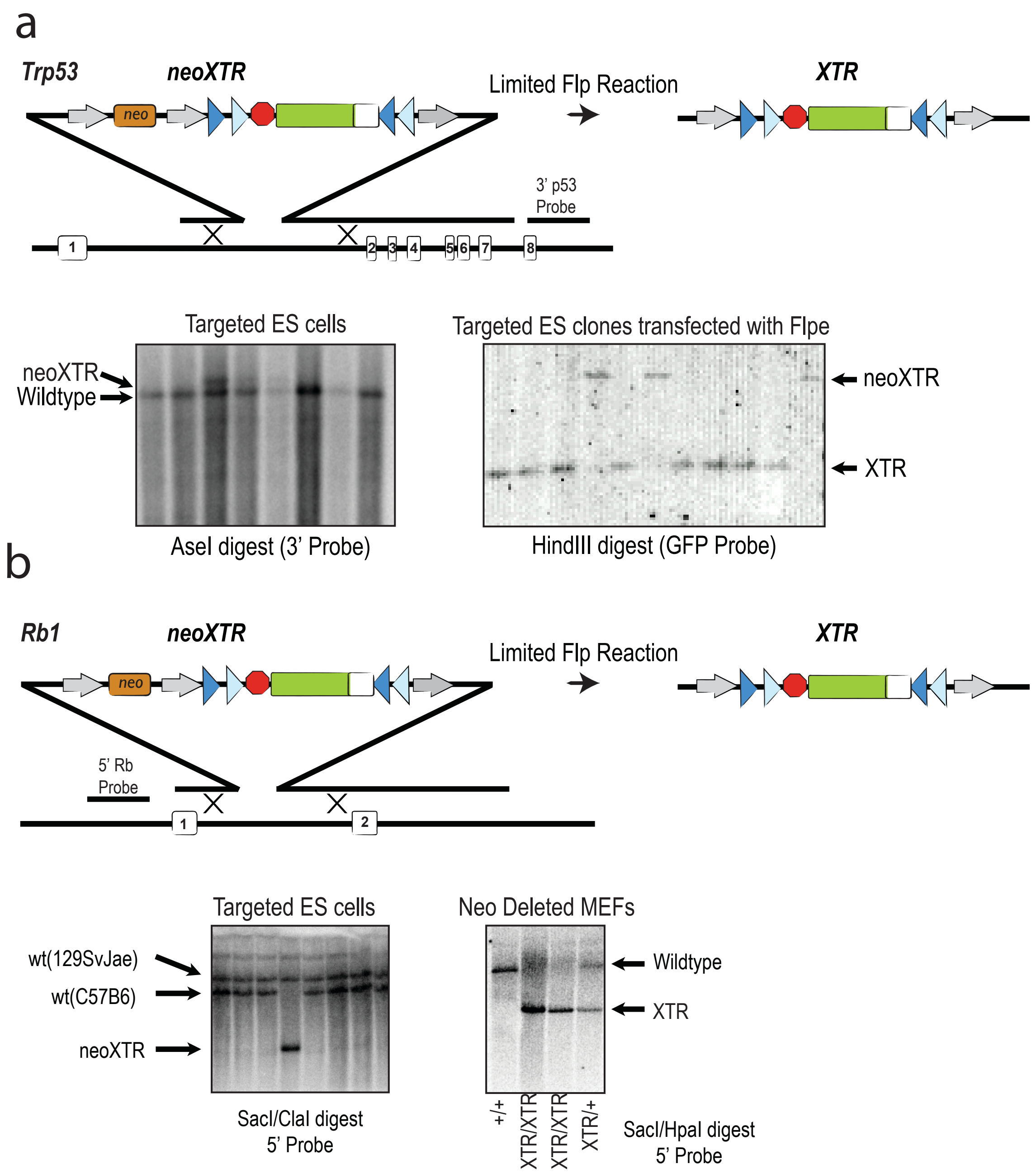

Supplementary Figure 2. Targeting of *Trp53* and *Rb1* with p53-NeoXTR and Rb-NeoXTR. Homologous recombination generates *neoXTR* alleles that require screening for limited Flp reactions that delete *neo*^{R} but retain *XTR*. Schematic of targeted *Trp53* (**a**) and Rb1 (**b**) alleles. Desired product of limited Flp reaction is depicted for each. Representative Southern blots identifying correctly targeted neoXTR alleles in ES cells (left) and successfully generated XTR alleles after limited Flp reactions (right).

a

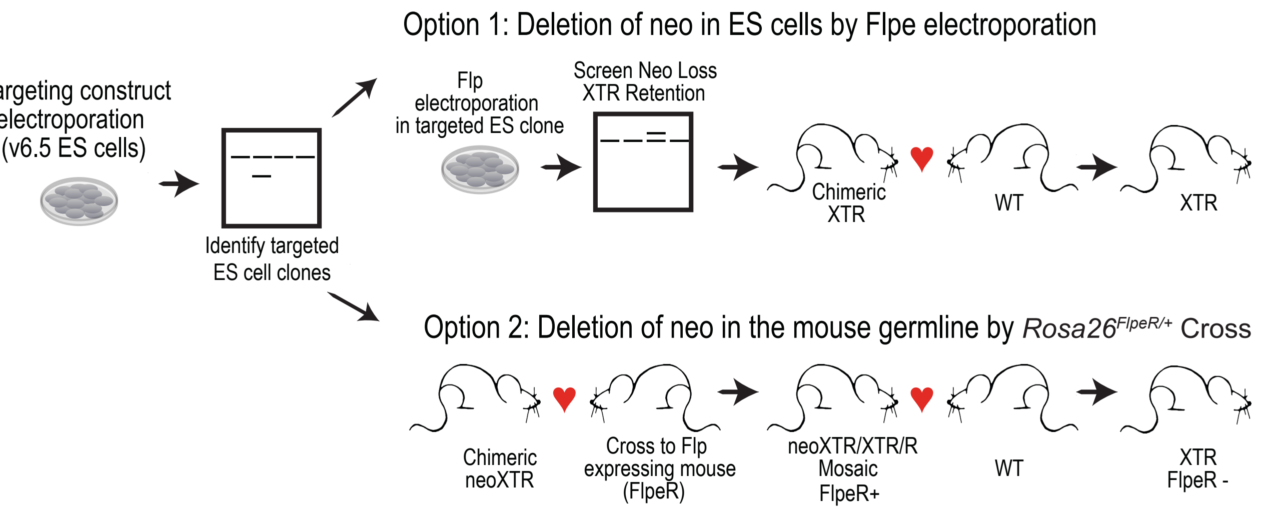

b

Method	$p53^{XTR/+}$	$Rb^{XTR/+}$	
Option 1:<br>Flpe electroporation	9/150	1/200	Number of ES cell clones that lost Neo but retained XTR /<br>Total number of clones analyzed
Option 2:<br>*Rosa26*$^{FlpeR/+}$ Cross	5/26	6/12	Number of pups that lost Neo but retained XTR and lost FlpeR allele /<br>Total pups that lost FlpeR allele

Supplementary Figure 3. Removal of *Neo*^{R} by limited Flp reaction. We targeted *neoXTR* alleles to both *p53* and *Rb* loci through standard ES cell electroporation and selection techniques and verified targeted integration by Southern blot (Supplementary Fig. 2). Correctly targeted clones harbor the *neoXTR* allele and strong expression of *neo*^{R} would likely interfere with the normal expression of the endogenous host gene, a phenomenon known as promoter interference. To avoid this, we engineered an additional *FRT* site 5' to the neomycin resistance gene in the *neoXTR* targeting vector (Supplementary Fig. 1). The resulting 3-*FRT* arrangement allows screening for loss of *neo*^{R} but retention of XTR. (**a**) For both p53 and Rb loci, we successfully established mouse lines that specifically lost the *neo*^{R} gene but retained the *XTR* allele either by electroporation of a Flp expression plasmid and re-cloning of ES cells, or by crossing *neoXTR* animals to germline Flp-expressing (*Rosa26*^{Flpe}) mice. In each case, ES cells or pups that lost the *neo*^{R} but retained *XTR* were identified by PCR and validated by Southern blot (Supplementary Figure 2 and not shown). (**b**) Table summarizing the relative efficiency of limited Flp reactions in both ES cells and the mouse germline.

Western Blots Related to Figure 1g

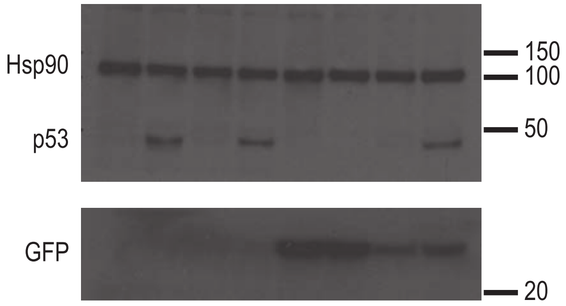

Western Blots Related to Figure 1f

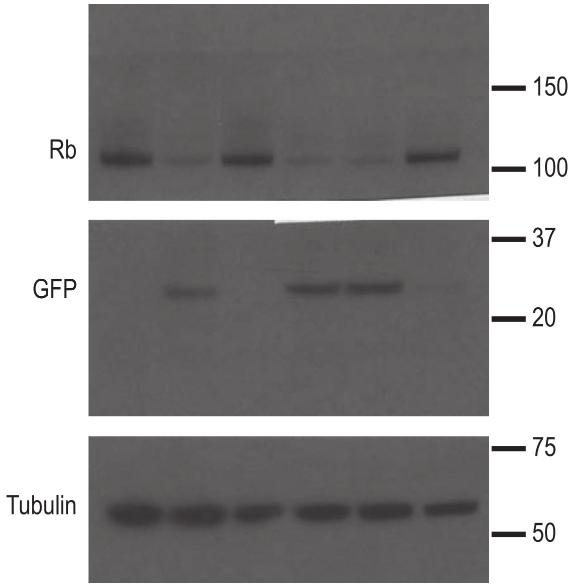

Western Blots Related to Figure 3c

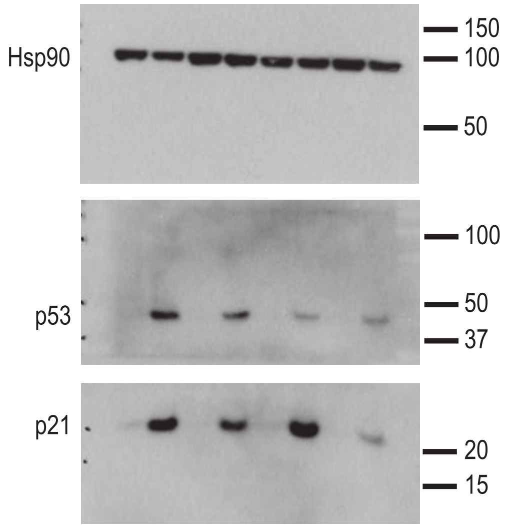

Western Blots Related to Figure 3d

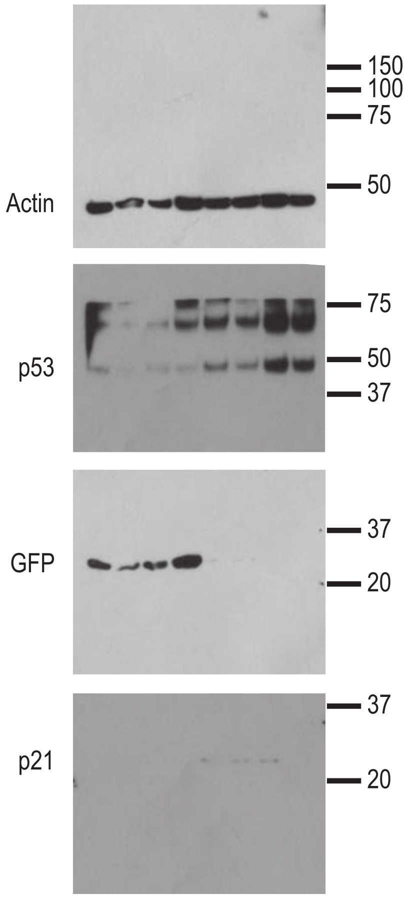

Supplementary Fig. 4 Full Western blots
